# Ethical considerations for the use of anti‐amyloid immunotherapy in patients with early Alzheimer's disease

**DOI:** 10.1002/alz.13795

**Published:** 2024-03-25

**Authors:** Susanne J. van Veluw, Michael J. Young

**Affiliations:** ^1^ Department of Neurology Massachusetts General Hospital Harvard Medical School Boston Massachusetts USA

Dear Editor,

Recently, the US Food and Drug Administration (FDA) approved two anti‐amyloid antibodies for the treatment of patients with early Alzheimer's disease (AD), with a third expected to be approved soon.^1^ These drugs are designed to remove amyloid‐β peptides from the brains of patients in the early stages of AD to slow down cognitive decline. The use of anti‐amyloid immunotherapy has raised several ethical issues, mostly centered around the dimensions of beneficence, nonmaleficence, and informed consent. We consider another potential area of concern related to the principle of human dignity.

The modest benefits and associated risks of recently FDA‐approved anti‐amyloid antibodies aducanumab and lecanemab^2^, ^3^, ^4^ have generated reluctance among some clinicians in prescribing these drugs to individuals with cognitive impairment, who may not fully grasp the nuances of potential benefits and harm. Due to ongoing scientific progress, it is increasingly likely that anti‐amyloid immunotherapies will become more effective in slowing down cognitive decline, be safer, and lead to better long‐term outcomes.^5^ In this scenario, are there other ethical objections to the routine use of these interventions worth considering?

We argue that there is still reason to pause on deontological grounds. A motivation for seeking anti‐amyloid immunotherapy, despite known risks, is the fear of losing one's dignity. We contend that routinely offering anti‐amyloid immunotherapy based on this motivation is questionable. Several moral frameworks, such as forms of deontological ethics, virtue ethics, and certain cultural and religious worldviews, maintain that human dignity is inherent to all human beings and not clearly contingent upon cognitive abilities or any external factors.^6^ According to this view, cognitive impairment or any reduction in cognitive abilities does not diminish a person's inherent dignity.

If preserving someone's dignity as a motivation to use anti‐amyloid immunotherapy were to be widely adopted in the public narrative, this may inadvertently result in a shift in collective thinking about the value of individuals with compromised cognition. If the prevailing justification for pursuing such interventions becomes the preservation of dignity, there is a hidden risk that those who choose not to pursue these interventions—for whatever reasons—may be tacitly regarded as having inferior worth and lower dignity than others. The presumption that compromised cognition categorically entails a loss of dignity is also at odds with the medical ethic of care, which supports difference and upholds respect for all persons, regardless of their (in)capacity.^7^

One objection might emphasize that the term “dignity” is to be understood differently in these contexts. Patients with AD are sometimes treated in undignified ways and may animate fear of cognitive decline. The dignity with which one is treated ought not to be linked to one's cognitive capacities in determining a person's moral status. Instead, dignity should be based on an individual's inherent worth, regardless of their cognitive status. Moreover, while expressions of personhood may indeed change with shifting cognitive capacities, intrinsic dignity is still preserved throughout the course of disease.^8^, ^9^

A second objection could be that there are indeed other motivations to seek anti‐amyloid immunotherapy, including the desire to preserve autonomy and independence, at least temporarily. These are reasonable ethical considerations for the use of these interventions, as they do not risk implicitly imposing value judgements on others. When considering these factors, one must carefully weigh potential benefits against associated risks and burdens, including the possibility that autonomy and independence may unexpectedly be detrimentally impacted rather than safeguarded following intervention.

## 1 CONCLUSION

As suggested by the appropriate use recommendations for lecanemab, the decision to pursue anti‐amyloid intervention or not should be made through ongoing conversations involving patients, clinicians, and their caregivers.^10^ These discussions should not only include nuances regarding the benefits and potential harms of the treatment but also a conversation related to the patient's values and motivations for seeking intervention.

Moreover, in parallel with investing resources and funding in further improving the effectiveness and safety of anti‐amyloid immunotherapy, efforts should be directed toward the system infrastructure that cares for cognitively impaired elderly individuals. This ensures they continue to receive care in the most dignified ways, regardless of whether one chooses to receive a given intervention or not.^8^

Finally, ongoing conversations in the public domain regarding the treatment of individuals with cognitive impairment should consider the wider implications for our collective notion of human dignity when invoking preservation of human dignity as a motivation to seek novel intervention. These discussions should ideally include the various stakeholders in this debate, such as patients, their caregivers and clinicians, pharmaceutical and biotechnology companies, the Alzheimer's Association, professional society organizations, scientists, policy makers, and neuroethicists.

## CONFLICT OF INTEREST STATEMENT

Dr. van Veluw has consulted for Biogen and Eisai and her lab receives research support from Therini Bio and Sanofi. Dr. Young has nothing to disclose. Author disclosures are available in the supporting information.

## Supporting information

Supporting Information
